# Resource-Constrained Onboard Inference of 3D Object Detection and Localisation in Point Clouds Targeting Self-Driving Applications

**DOI:** 10.3390/s21237933

**Published:** 2021-11-28

**Authors:** António Silva, Duarte Fernandes, Rafael Névoa, João Monteiro, Paulo Novais, Pedro Girão, Tiago Afonso, Pedro Melo-Pinto

**Affiliations:** 1 Algoritmi Centre, University of Minho, 4800-058 Guimarães, Portugal; asilva@algoritmi.uminho.pt (A.S.); rafael.accn@gmail.com (R.N.); joao.monteiro@dei.uminho.pt (J.M.); pjon@di.uminho.pt (P.N.); or pmelo@utad.pt (P.M.-P.); 2 Bosch Company, 4700-113 Braga, Portugal; pedro.girao@pt.bosch.com (P.G.); tiago.afonso@pt.bosch.com (T.A.); 3 Department of Engineering, University of Trás-os-Montes and Alto Douro, 5000-801 Vila Real, Portugal

**Keywords:** autonomous driving, deep learning methods, LiDAR scanners, 3D object detection, onboard inference, quantisation methods

## Abstract

Research about deep learning applied in object detection tasks in LiDAR data has been massively widespread in recent years, achieving notable developments, namely in improving precision and inference speed performances. These improvements have been facilitated by powerful GPU servers, taking advantage of their capacity to train the networks in reasonable periods and their parallel architecture that allows for high performance and real-time inference. However, these features are limited in autonomous driving due to space, power capacity, and inference time constraints, and onboard devices are not as powerful as their counterparts used for training. This paper investigates the use of a deep learning-based method in edge devices for onboard real-time inference that is power-effective and low in terms of space-constrained demand. A methodology is proposed for deploying high-end GPU-specific models in edge devices for onboard inference, consisting of a two-folder flow: study model hyperparameters’ implications in meeting application requirements; and compression of the network for meeting the board resource limitations. A hybrid FPGA-CPU board is proposed as an effective onboard inference solution by comparing its performance in the KITTI dataset with computer performances. The achieved accuracy is comparable to the PC-based deep learning method with a plus that it is more effective for real-time inference, power limited and space-constrained purposes.

## 1. Introduction

The rapid development of computational power brought by high-end GPUs allowed for deep learning algorithms’ increased importance in object detection tasks for a wide variety of domains and, more particularly, in autonomous driving using Light Detection And Ranging (LiDAR) data. This represents tremendous gains in detection efficiency, both in terms of accuracy and inference speed. However, deep learning methods are typically computationally expensive and, therefore, demand high-end server graphics processing units (GPUs). As deep learning methods go deeper in an attempt to extract more and more meaningful features, the more computationally expensive they become. The cheap availability of GPUs allied to their capacity to train networks in reasonable periods by taking advantage of their parallel architecture results in high performance and real-time execution of deep learning models. However, their use as edge devices instead of server machines is still impractical due to a critical requirement: power efficiency. Moreover, in a vehicle, onboard computational resources exhibit limited execution time and space. Although the scientific community widely adopted network optimisation and compression techniques to cope with these limitations, research studies in applying Convolutional Neural Network (CNN)-based models in resource-constrained devices [1,2,3] show that accuracy degradation is expected to be relevant and can affect the capacity of models detecting and classifying target classes. However, 3D object detection models addressed in the literature takes as input point clouds and are known for being more complex. They have a deeper pipeline and process a larger amount of data. For instance, typically a point cloud comprises between 100 k–120 k points [4], where each point contains data related to euclidean distance and signal reflection, i.e., 128 bits for translating each point information. Although solutions in the literature differ in the design of its pipeline, they share the same type of backbone, i.e., a stage responsible for extracting features from input data, which are based on CNN, and therefore due to the amount of data and arithmetic operations is the most computational demanding stage of the pipeline. This paper focuses on how computing demand processes such as CNN-based operations can be deployed in resource-constrained devices, proposing a methodology that covers all the steps needed, such as hardware and software generation and model optimisation, aiming at providing a 3D object detection model able to offer real-time inference without significant penalties on the performance metric precision. The contributions featured in this work are as follows:

- Demonstration of how the inference step of Object detection models and processing point clouds can be implemented in an edge device limited in size and power computation;
- A fine-tuning process and respective implications of deep learning model hyperparameters in meeting edge device application requirements;
- Application and analysis of different compression methods and their impact on the model performance;

To the best of our knowledge, this paper is the first attempt to deploy an onboard point cloud-based 3D detection model, which requires greater computation power and resources that hybrid FPGA-CPU boards do not typically have, for detecting, localising and classifying objects in point cloud data.

The paper is organised as follows: Section 2 describes related work regarding systems for object detection and hardware platforms for their deployment. Section 3 presents a four-step methodology used to select, train and fine-tune a deep learning model for deployment in a hardware device. In this section, we present the selected model regarding its deep learning components, the details about the architecture of the target hardware device, and the implementation of hardware and software components. The process of deploying the object detection model to the edge device takes place in Section 3.3, where the details about the compression and quantisation processes are also given. Section 4 presents benchmarks of the floating-point and integer versions of the models previously presented in Section 3.3.2. In Section 4, the comparison of the quantitative test of the floating-point and integer versions of each model configuration is presented. Finally, Section 5 presents the conclusions and future work considerations.

## 2. Related Work

In recent years, we have witnessed the rapid development of deep learning methods. CNN-based deep learning algorithms have been mainly applied for applications such as object detection, applications in medical image analysis, and speech recognition [5] but its applicability is wider, as proven by recent novelty works. For instance, in [6] the authors proposed an algorithm based in CNN to improve the security performance of IoT-Enabled Healthcare Networks.

Regarding the object detection in point cloud data, models addressed in the literature have increasingly improved their detection capabilities. Generally, models in the literature have been positioned in two categories: 3D and 2D CNN-based approaches, where different data representation, backbone networks, and multi-scale feature learning techniques might be adopted [4].

The first methods typically rely on volumetric representation to discretise the point cloud. Examples include VoxelNet [7], SECOND [8], F-PointNet [9], Point $A^{2}$-free [10], Point $A^{2}$-anchor [10], HVNet [11], and then 3D convolution-based methods are performed to produce object class prediction, bounding box regression, and orientation classification. Typically, these methods demand more computationally expensive processes either because they apply the expensive volumetric representation of the point cloud or the 3D-based heavy convolutions. Although models have proposed solutions to address this topic by introducing methodologies or techniques to reduce complexity [12,13,14], typically, these methods present lower inference speeds when compared with 2D CNN-based [4]. This limitation makes it difficult to adopt them for real-time applications.

Models that shift the 3D-based heavy convolutions by 2D convolutions, such as LaserNet [15], VeloFCN [16], MV3D [17] or PointPillars [18], usually opt by compacting the information into a 2D projection or organise point clouds in Pillars [18] as a means to reduce the high computational cost of representing and processing 3D LiDAR data. Although these methods present lower inference time results and thus facilitate their application for real-time purposes, they reduce their detection capabilities by introducing an information loss. It shows that there is always a trade-off between accuracy and inference time results.

Meeting application requirements in driverless vehicle setups is a challenging task, especially when 3D object detection models applied in point cloud data are only studied in powerful server nodes, as it is not reasonable to put such a power source and space demand in a vehicle setup. Thus, dedicated hardware accelerators have gained increased importance mostly due to their compactness, robustness, flexibility and performance. A study was conducted to compare different dedicated hardware accelerators for vision-based navigation applications, namely FPGA, CPU, GPU, and DSP [1]. It shows that FPGAs deliver higher performance per watt than micro-controllers or GPUs, which is advantageous for limited power supply and space-constrained setups. Moreover, GPUs demand more power supply resources when compared with FPGAs and CPUs in the same conditions of operation and inference accuracy. However, FPGAs have better performance compared with CPUs for deep learning purposes. Although FPGAs have gained a substantial space in the scientific community as latency and energy efficiency inference accelerators for deep and machine learning applications in real-time inference, it has limited resources compared with high-end GPUs [1]. These limitations forced the need of reducing the network size by applying network compression techniques, such as quantisation, pruning, weight reduction [1], or design methodologies for enhanced architectures in terms of efficiency, such as loop unrolling, memory configurations and utilisation, strategies for mapping network into hardware, optimised data flows, and computing unit designs [19]. Given the nature of some operations found in 3D Object Detection models, such as prepossessing (data structuring) and post-processing - classification heads and Non-Maximum Supression operations (nms) for instance, hybrid computing devices comprising FPGAs ans Systems-on-Chips are preferred for implementing deep learning algorithms for edge devices in several applications [1,20,21,22], speed and energy being the main criteria for target board selection. These works leverage the parallelism nature of these accelerators to deliver real-time inference for applications.

Qiu et al. proposed the first parameterised and runtime configurable hardware architecture compatible with several networks triggering the forthcoming commercial FPGA-based accelerators, such as HADDOC2 [23], DNNWEAVER [3], Hls4ml [2], and Vitis AI [24]. These frameworks provide solutions to compress networks, reducing the memory footprint and inference time, offering automatic flows for data quantisation and pruning, and compilation for mapping a CNN model to the hardware platform. The differences between the frameworks can be found in the following features: (1) model input format; (2) deep learning operators; (3) optimisation and compression techniques supported; and (4) supported hardware devices.

**Figure 1:**
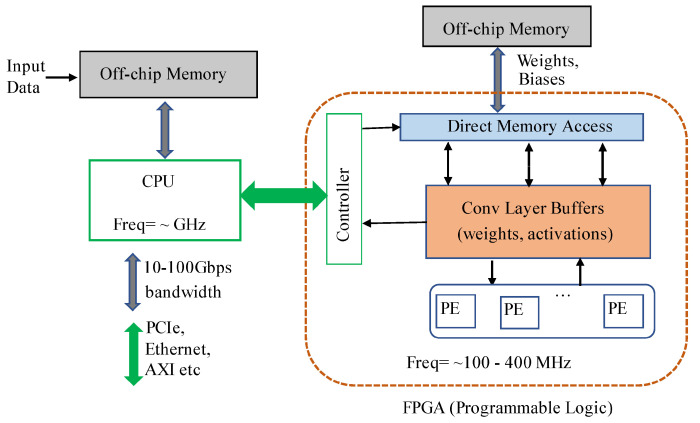
Traditional architecture of hybrid FPGA-CPU based inference solutions [1].

(1) Onto model input format support, both Hls4ml [2], and Vitis AI supports a wide variety of higher-level, familiar deep learning frameworks such as Keras, TensorFlow, Caffe and Pytorch. On the other hand, DNNWEAVER [3], and HADDOC2 [23] support fewer off-the-shelf deep learning frameworks by only supporting Caffe and Tensorflow frameworks, respectively.
(2) All frameworks provide automatic workflows for mapping deep learning model operators. HADDOC2 use direct hardware mapping (DHM) for implementing Caffe deep learning approaches onto FPGAs. Deep learning entities are associated with private resources to maximise parallelism and neurons in a layer mapped on the target device to take interneuron parallelism. DNNWEAVER takes deep learning models coded in Tensorflow and maps them to a macro dataflow instruction set architecture (ISA). For this purpose, they use a translator component for converting deep learning specifications to their macro dataflow ISA. In Hls4ml, an automatic workflow is used [25] to represent deep learning operators and convert deep learning models. Then, the converted model is deployed into the FPGA firmware. Also, Vitis AI uses automatic workflows to convert customised deep learning tasks or complete models to run on an accelerator placed on the FPGA fabric called Deep Learning Processing Unit (DPU). DPUs are scalable to fit various Xilinx platforms and are customisable to meet performance application-specific needs.
(3) Optimisation and compression techniques are also supported by projects described herein to achieve high-performance inference times without sacrificing too much accuracy. HADDOC2 uses fixed-point numerical representations to describe CNNs variables and multiplications with logic elements to optimise models. DNNWEAVER compiler uses an optimisation algorithm that tiles, schedules, and batches deep learning operators to maximise data reuse and optimise target FPGA memory and other resources. In Hls4ml, the deep learning model optimisation is made by reusing the resources of the inference operation and using compression techniques such as pruning and quantisation in binary and ternary precision. Vitis AI enables the optimisation of models through AI Quantiser and AI Optimiser. AI Quantiser supports pruned and unpruned model quantisation, calibration, and fine-tuning, while AI optimiser aims at pruning redundant connections in neural networks and reduces the overall required operations. Moreover, the Vitis AI framework allows manipulating the DPU engine to meet the deep learning model resource requirements and model compression to reduce model complexity.
(4) The compiled model in HADDOC2 can be output to any FPGA device with tools supporting VHDL 93. DNNWEAVER model result is a synthesisable accelerator that matches deep learning model needs and, at the same time, provides performance and efficiency gains for any target FPGA. Hls4ml framework deploys the converted deep learning model onto any target FPGA device. Adopting the previously mentioned engines requires the customisation of the hardware design regarding the network and application, hampering its application in real-world applications. In this context, the Viti Ai proposed and hardware IP Xilinx Deep Learning Process Unit (DPU) [26] that can be easily adapted for the most well-known applications for several purposes, for instance, 2D or 3D object detection, segmentation, etc. providing support for a wide range of hardware platforms, such as Alveo cards, Ultra scales FPGA, and embedded platforms, while providing APIs for easily scheduling jogs for DPUs while promotes an efficient interaction processing system (PS) and a Programmable Logic (PL) unit, both depicted in Figure 1. Moreover, it also supports various frameworks for deep learning (Caffe, Tensorflow and Pytorch) and their basic functions.

PS side refers to the CPU, typically responsible for the pre- (e.g., data cropping, data transformations, and others) and post-processing (multi-head stage for location, classification and bounding box regression, and others) tasks of the deep learning pipelines and task scheduling; and external memory (e.d. DRAM), where input data, bias, weights and PL instructions are stored. The PL side refers to the FPGA chip comprising the processing elements (PE) for executing convolution-based arithmetic operations of the most complex operations of the pipeline, i.e., convolutions and fully-connected layers; and internal memory, such as BRAMs for holding data from activation layers as well filter weights.

## 3. Methodology

**Figure 2:**
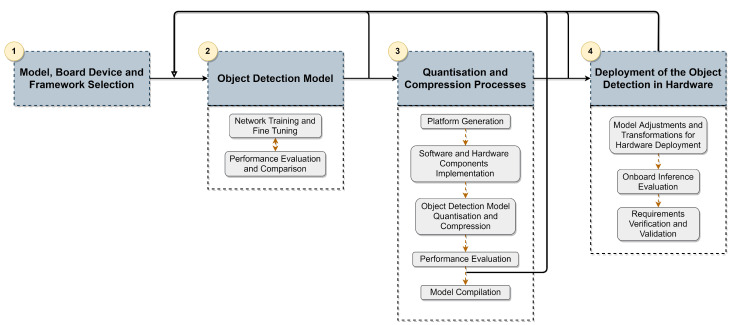
Methodology used for the deployment of the object detection model in the hardware device.

To deploy the deep-learning-based model in a hardware device, we used the four-step methodology depicted in Figure 2. (1) The model selection was based on the literature review of existing models for 3D Object Detection, and then compatible frameworks and corresponding hardware devices that accommodate the selected model were studied.

After this step, the selected model was subjected to a training and evaluation pipeline (2), where several optimisations were taken to improve accuracy metrics while granting the inference time requirement. This intense pipeline was performed in a server-side node (Intel Core i9 with 64 GB RAM and a Quadro RTX 8000 GPU). The proposed workflow follows an iterative approach, where the model is fine-tuned, and steps of training and evaluation are repeated whenever required. The evaluation and comparison process is conducted using KITTI benchmarks on the validation set in the before-mentioned server node. In conclusion, this workflow ensures that our model meets the application requirements and achieves the best possible accuracy. From this process, a set of candidate network configurations are selected for the next step.

Once the workflow of step (2) is completed, a quantisation and compression phase (3) is carried out, where the models obtained in the previous step are optimised and compressed. In this phase, the performance of the optimised and compressed version of the 3D object detection model is analysed with regard to accuracy degradation and speed improvement. The model compilation phase only occurs when an optimal balance between the metrics is achieved; otherwise, the workflow restarts at stage (2).

After the model compilation, adjustments and transformations are performed to prepare the models for respective deployment in the hardware devices (4). Next, the inference is performed in the KITTI validation set to evaluate the model’s accuracy and validate the inference time metric. The model’s accuracy evaluation provides insights about the accuracy degradation regarding the quantisation and compression phase, while the validation of the inference time allows granting the onboard inference time lower than 100 ms for meeting application requirements. Thus, steps (2) and (3) can be repeated to ensure satisfactory accuracy performance while respecting inference time requirements. The following section provides details about the selected deep learning model’s architecture and the parameters used in the fine-tuning process.

### 3.1. Object Detection Model

**Figure 3:**
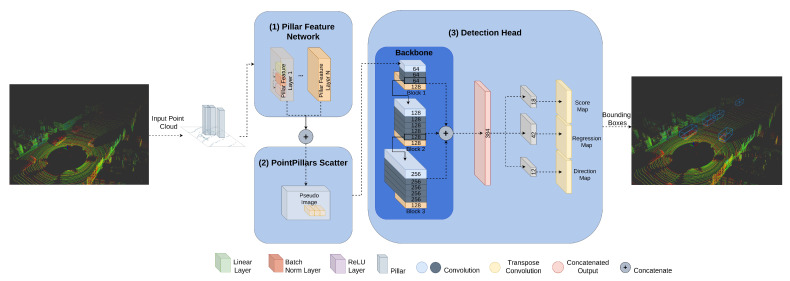
Object Detection Network pipeline.

We opted for the model PointPillars [18] for deployment in the hardware device since this model relies on 2D dense convolutions to the detriment of more complex 3D convolution-based features extraction networks and achieves high-quality detection with low inference time. As reviewed in [4], this model ensures an optimal trade-off between accuracy and inference time. As depicted in Figure 3, it consists of three main components: (1) Pillar Feature Network, (2) PointPillars Scatter and (3) Detection Head.

The first component, (1) Pillar Feature Network, the local feature extractor, receives as input a set of pillars and encodes a set of features that are (2) scattered back to a 2D pseudo-image.

Then, the 2D Backbone extracts features from this image-like representation that are used by the (3) Detection Head, which in turn performs a set of 1 × 1 convolutions to predict object class and bounding box offsets and direction. This 1 × 1 convolution replaces the original PointPillars Single Shot Detection Head. In this work, the backbone is represented as a set of blocks BLC, in the form $blc_{1},blc_{2},\ldots ,blc_{m}$, where m≥1. Each block $blc_{j}\in BLC,j\leq m$, is represented by (n,F,U,S). The element *n* represents the number of convolutional layers in $BLC_{j}$. The set of convolutional layers *C* in $BLC_{j}$ is described as a set $c_{1},c_{2},c_{3}\ldots c_{n}$, where n≥1. *F* represents the number of filters of each $c_{i}\in C,i\leq n$, *U* is the number of upsample filters of $c_{i}$. All upsample filters are the same, and their respective outputs are concatenated. *S* denotes the stride in $c_{1}$. If S>1 we have a downsampled convolutional layer ($c_{1}$), represented in Figure 3 as a light blue box. This layer is followed by several convolutional layers ($c_{i}$, such that i>1), represented in Figure 3 as dark blue boxes. After each convolutional layer, BatchNorm and ReLU layers are applied. Finally, a set of 1 × 1 convolutions $C1x=c1x_{1},c1x_{2},\ldots ,c1x_{k}$, where k=3, is applied. Our baseline network for representing each block is depicted in Figure 3, where we use three blocks, and each block is represented as follows:

- $blc_{1}=\left(3,64,128,2\right)$;
- $blc_{2}=\left(5,128,128,2\right)$;
- $blc_{3}=\left(5,128,128,2\right)$.

#### 3.1.1. Network Training and Fine-Tuning

All models were trained on the KITTI dataset and evaluated on the KITTI benchmarks for 3D object detection. We used two methodologies for the number of training epochs. In one methodology, used by [8], models are trained for 160 epochs, and in another for 300 epochs. The initial learning rate, exponential decay factor and the decay epoch methodology remain the same as referred in [8]. Also, the same values for decay weight, beta1 and beta2, were used. All detection results are measured using the official KITTI evaluation detection metrics called average precision (AP) for a bird’s eye view (BEV), 3D and 2D perspective. The training dataset was split using the approach adopted in [17], which consists in splitting the provided 7481 training examples into a training set of 3712 samples and an evaluation set of 3769 samples. Thus, the benchmarks provided here are based on the evaluation set only.

For all experiments, we selected three categories - car, pedestrian and cyclist as our target classes. The model detailed in [18] generates two separate networks, one for predicting cars and another for pedestrians and cyclists, which can have a high computational cost, especially when running it in the edge device (few resources need to cope with two parallel models). Another problem is granting that both models process the point cloud and generate output bounding boxes simultaneously or within a short time difference and in less than 100 ms. Thus, we train these three instances in a one-single network.

For the fine-tuning process, a random search methodology was employed to search for the best hyper-parameter that ensures a better balance between accuracy and inference time. Next, We will describe the hyper-parameters used for the fine-tuning process. The resulting set of experiments and respective network configurations are then summarised in Section 3.1.2.

**Detection Head Stride and Filters.** Typically, cyclist and pedestrian detection are the more difficult task in 3D object detection using the KITTI dataset, since these instances have fewer points to describe their shape. Moreover, using large strides results in fewer pixels being analysed, which makes detection even more difficult. For this purpose, we optimised Detection Head block strides (*S*), filters (*F*) and upsampling filters (*U*) for better capturing of point cloud features for these classes. The idea herein is to find the most appropriate balance between these parameters and, at the same time, granting the onboard inference time requirement. As such, we used four Detection Head configurations as depicted in Table 1. For all configurations, stride one in the first block ($blc_{1}$) was used for capturing more features. Although it generates a higher feature map, it increases the memory usage and consequently the inference time. Thus, to ensure the inference time requirement, we chose a lighter version of Detection Head, where a small number of downsampling and upsampling filters are used. The other two versions keep the same stride for the first block while increasing the number of upsampling and downsampling filters.

**The number of Sampling Instances.** The strategy followed here was to search an optimal number of sampling instances. These classes are randomly selected and placed into the current point cloud used in the training stage. Our focus herein is to soften the KITTI dataset imbalance issue. In our experiments, we use the original configuration as mentioned in Section 3.1, and three experiments were conducted as demonstrated in Table 2.

**Point Cloud Range.** We receive an unordered set of points $PC=\{p_{1},p_{2},p_{3}\cdots p_{n}\}$, where n≥0 and each point *p* is represented as $\left(p^{x},p^{y},p^{z},p^{r}\right)$, where $p^{x},p^{y}$ and $p^{z}$ correspond to coordinates in the three-dimension cartesian axis and $p^{r}$ is the reflectance value provided by the LiDAR sensor. A point cloud range $PC_{R}$ is a tuple (L,H,W), where *L* consists of $\left(x_{min},x_{max}\right)$, *H* consists of $\left(y_{min},y_{max}\right)$, and *W* consists of $\left(z_{min},z_{max}\right)$. We denote a point cloud subset with respect to $PC_{R}$ as $PC_{R}=\left\{p_{i}:p_{i}\in PC,x_{min}\leq p_{i}^{x}\leq x_{max},y_{min}\leq p_{i}^{y}\leq y_{max},z_{min}\leq p_{i}^{z}\leq z_{max}\right\}$. Point cloud range directly affects the model detection range and thus limits its detection range. In our research, we conducted a study to represent the location of ground truth objects for all frames in the KITTI dataset frame. As discussed in Section 4 for example, in cars, it is possible to verify in terms of depth information that most ground truth instances are between the 0 and 70 m, and after 70 m from the LiDAR sensor centre, the number of instances drastically decreases. This can be explained by the fact that after this range, the number of points to describe objects shape is very few, making the task of detecting objects difficult. Thus, this experiment aims to find the optimal point cloud range where the detection range is not compromised. The point cloud ranges are depicted in Table 3.

**Pillar size.** The object detection model receives the points in $PC_{R}$ and discretises them in the *X*-*Y* axis thus creating a set of pillars $\left\{Pl_{1},Pl_{2},Pl_{3}\cdots Pl_{n}\right\}$, where where n≥0. Each Pl has a fixed size in $PC_{R}$ and it is represented by a tuple $S_{PL}=\left(l,h\right)$, where *l* is the length of the pillar along the *x* axis and *h* is the height of the pillar along the *y* axis. The Pillar size has a direct impact on model accuracy and inference time. Increasing the Pillar size can result in too much data having to be encoded and consequently randomly sampled, which leads to information loss (maximum number of points per Pillar is set for computational saving purposes as referred in Section 3.1). On the other hand, reducing the Pillar size can increase the number of non-empty Pillars, increasing memory usage and, consequently, inference time. In our fine-tuning process, three Pillar size configurations were used, as shown in Table 4.

**Number of Pillars.** The maximum number of Pillars is defined to explore the KITTI dataset sparsity problem, since most Pillars will be empty. Using a large number of Pillars can result in most of them being filled with zeros (to create a dense tensor as mentioned in [18]), making it inefficient for inference time purposes. Based on the distribution of the number of points per Pillar in the KITTI dataset, a max number of points is also defined. Table 5 shows the max number of points per pillar configuration used in our experiments.

#### 3.1.2. Performance Evaluation and Comparison

This section reports the set of experiments which results from the random search methodology used to achieve a better trade-off between accuracy and inference time performance metrics. The experiments and corresponding network configurations are depicted in Table 6. PointPillars settings and their results are also provided to understand the impact of producing a model optimised to produce three-class output rather than separating it into two distinct networks (one for cars and another for pedestrians and cyclists).

The following Table 7, Table 8 and Table 9 provide the results of experiments of Table 6 in terms of AP for three difficulty levels (Easy, Moderate and Hard) and different Intersection over Union (IOU) thresholds, according to KITTI benchmarks. For cars, the IOU is 70%, while for pedestrians and cyclists, it is a required IOU of 50%.

As demonstrated in the aforementioned results, there is a cost in terms of AP for three-class trained models compared with PointPillars-separated networks (a standard literature practice on KITTI benchmarks). During training, gradients are affected by all those instances, which leads our models to lose the specialisation for prediction. These costs are more evident when analysing the inference times (results in Table 10) since, in general, experiments perform worst in this metric compared with PointPillars. The stride one in the first RPN block and the substantial increase in the number of generated anchors during the RPN detection phase can explain this difference. While the point cloud range in our networks is the same across all instances, PointPillars generates anchors in a shorter range for pedestrian and cyclist classes, which substantially reduces the number of generated anchors. Moreover, placing stride one in the first RPN block results in a higher feature map and consequently a decrease in the model inference speed. An example of this is the network of experiment 2, which improves the AP results compared with PointPillars by increasing the number of filters and upsample filters and applying stride 1 to the first RPN block, but at the cost of increasing inference time.

To solve this limitation, we moved towards our efforts to change the training procedure by increasing the number of sampling instances and the number of training epochs. Just increasing the number of sampling examples brought improvements, especially for rare classes such as pedestrians and cyclists. The results were quite prominent by combining them with an increased number of training epochs (experiment 7). Although this is true, experiment 6, where we use the *SI* configuration with the highest number of sample instances while maintaining 160 training epochs, demonstrates no practical improvements in terms of AP compared with the network of experiment 5.

Also, we explore different point cloud configurations by changing the Pillar sizes, Point Cloud ranges, and the total number of Pillars. As expected, the results for $S_{PL25}$ were worse, while $S_{PL16}$ and $S_{PL5}$ pillar sizes present better results, but they are very similar. However, $S_{PL5}$ implicates more memory resources since more Pillars are generated. In the $S_{PL16}$ configuration, the point cloud from an HDL-64E (sensor used in KITTI) generates 4 k–9 k non-empty pillars, and $S_{PL5}$ generates even more, which drastically affects inference time (more Pillars need to be filled with zeros). On the other hand, increasing the pillar size too much, such as $S_{PL25}$, leads more Pillars to hold too much data (max number of points of 100 is defined per Pillar) to fit the defined PointPillars tensor, as mentioned in Section 3.1. Consequently, the data is randomly sampled to cope with the max number of points, which leads to information loss.

Although the results of the PointPillars separated networks seem promising in terms of the trade-off between accuracy and inference speed, as mentioned before, this literature standard practice is impractical for onboard hardware device inference. Thus models herein were produced to cope with this limitation. After analysing the experiments performed, we selected the networks of experiments 1, 7, and 11 as candidates for the next phase. Generally, these models outperform the PointPillars original networks concerning mAP but at the cost of increasing the inference time, as shown in Table 10. Although results of inference time meet the application requirements, these values need to be subject to in-depth analysis for FPGA onboard inference purposes since these values have a small margin compared with the reference value (100 ms) and can suffer fluctuations during inference. The memory usage, the processes running in the background, and the number of objects of interest in a LiDAR scene can affect these candidates’ networks’ inference speed and lead to exceeding the reference value. In the next section, these networks will be subject to a depth analysis to understand the viability of their deployment in step 4. The next step of our methodology, called Quantisation and Compression Processes, will be described in the next section.

### 3.2. FPGA Inference—Platform Generation

The target device for the implementation of the above described deep learning network in hardware is the Zynq UltraScale+ MPSoC ZCU102 hardware platform from Xilinx, Inc. This device comprises several processors on a single chip, such as a real-time processing unit (RPU), which is a dual-core Cortex-R5F, an application processing unit (APU), which is a quad-core Arm Cortex-A53, and a graphics processing unit (GPU), which is a Mali-400, as well as logic blocks on the Programmable Logic (PL)-side of the chip for configuration of specific user operations. Summing up, the ZCU102 evaluation board supports high-level operating systems such as Linux, logic programming in the FPGA fabric and provides several peripherals (e.g., communication peripherals such as Ethernet or USB). All these features are crucial for the successful onboarding inference of the deep learning model and the consequent integration of our platform in the vehicle setup described in [27].

Regarding the programmable logic resources features, this platform offers 500 k logic Cells, 550 k CLB flip-flops, 2520 DSP slices, 24 GTH 16.3 Gb/s transceivers, up to 32.1 MB Block Memory (BRAM) in blocks of size 36 Kb each (a total of 900 BRAMs), 36 MB UltraRAM, and 8.8 MB Distributed RAM. Moreover, the PL side is equipped with a 512 MB DDR memory component. Moreover, the Vitis environment provides a programmable hardware engine optimised for convolutional neural networks, called a Deep Learning Processing Unit (DPU). This FPGA-based inference accelerator is implemented in the PL side of the target hardware board device, consisting of FPGA building blocks, such as multipliers, adders, and accumulators, which consumes part of the PL resources above-mentioned. This IP is configurable and requires specific instructions from a specialised instruction set, provided in the form of an xmodel file to implement a vast range of CNN architectures.

The DPU engine supports several deep learning operations, such as convolutions, pooling, activation functions, concatenation, deconvolutions, and others, and also allows the customisation of several parameters aiming at optimising resource usage, block performance in terms of energy consumption and inference time, as further analysed. Such parameters are DPU architecture (specified the level of parallelism), the number of DPU cores (up to four DPU cores can be instantiated in a DPU IP), RAM and UltraRAM usage (configures memory usage, high amount of memory offers higher flexibility in handling intermediate information, improving performance), and DSP cascade Length can be configured.

**Figure 4:**
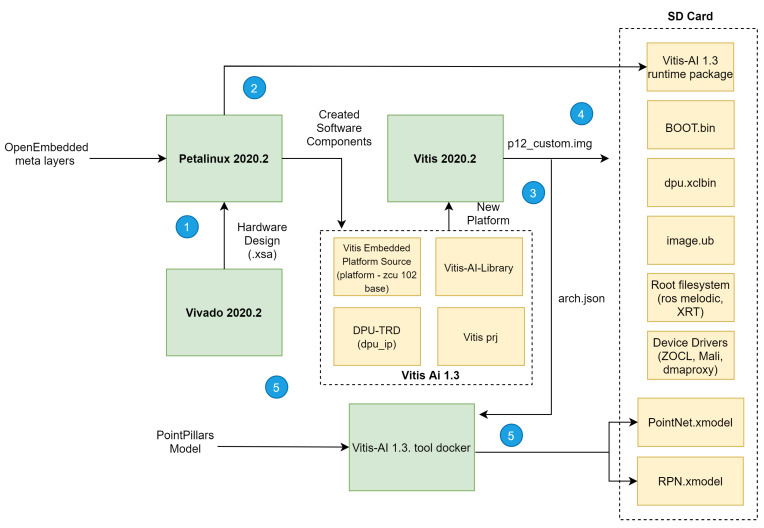
Hardware and Software implementation flow for inference.

For a proper deployment of our deep learning network targeting onboard inference, we set the methodology depicted in Figure 4 to implement both hardware and software required components. The methodology flow consists of five main steps: (1) building hardware design (.xsa and bitstream); (2) generation of the software components for the target device; (3) integration of both hardware and software components into a single platform; (4) building a platform project and an SD card image; and (5) adapting, optimising and compiling our deep learning model for onboard inference and building of an executable software to run on the target device with support for communications with devices external to the platform.

#### 3.2.1. Hardware Components Implementation

To implement the hardware design of our platform, an IDE from Xilinx called Vivado was adopted. For the efficient implementation of CNNs, a set of considerations were identified and followed to integrate this IP in our board correctly. First, direct connections between PL and PS sides must be provided, then memory locations for input point cloud, temporary and output data must be defined, and the interrupt connections between PL and PS sides.

**Figure 5:**
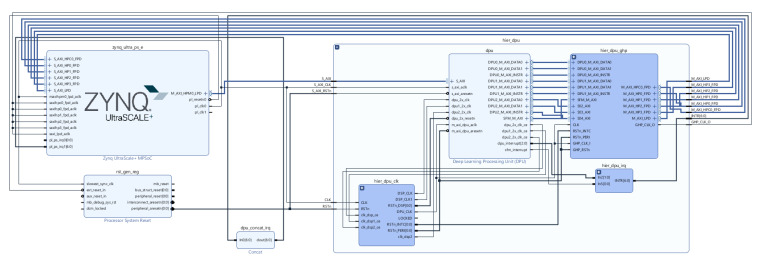
Hardware Design of DPU connected to the PS side.

DPU IP was imported and instantiated along with other IPs, as depicted in Figure 5. These IPs, such as Clock Wizard and Processing System, are instantiated in the PL side that communicates with the PS side, represented as the IP Zynq UltraScale+ MPSoC, via the AXI4-based PS-PL interface. The development of the PS side occurs during the phase of software development/phase 2 of the flow of development, depicted in Figure 5. Due to the DPU AXI slave interface, the registered address and address range must be assigned to the DPU IP. We set the minimum memory needed by the DPU to 16 MB. The device driver and device tree file addresses match those assigned in the previous step (Vivado). Figure 5 depicts the final hardware design of our platform.

After implementing and synthesising the hardware design, a bitstream file is generated, which can be exported to be used as the base hardware upon which the software is built in the following phase of the implementation flow, depicted in Figure 4.

In the setup overview showcased in Figure 1, we can see that the input data is obtained extranaly to the on-board, which might be a LiDAR sensor or a computer publishing point cloud for a ROS topic through the Ethernet port. The PS software accesses this data via the ROS node running that subscribed to such a topic and then inputs it to the DPU during inference.

#### 3.2.2. Software Components Implementation

During the second step of the Hardware and Software Implementation Flow for Inference, designated Software configuration, a Linux image is generated for an AArch64 hardware architecture. Our custom image must incorporate all resources, such as drivers, applications, and tools required to deploy deep learning networks on DPU. From the perspective of deep learning inference and external communication, the platform must provide Vitis AI Runtime (VART), Xilinx Runtime (XRT), ZOCL, and ROS packages. The VART tool was incorporated as a package in the yoc-to/petalinux flow, as it consists of a programming interface providing C++/Python APIs that can be invoked in our executable software for implementing accelerated AI inference on our hardware platform. This tool allows the developers to incorporate several functionalities in the executable software to execute kernels, as is the case of our DPU kernel, with support for multi-threading and multi-process execution. The most relevant functionalities are as follows: DPU kernel loading, which refers to tasks such as fetching DPU instructions and network parameters from the compiled deep learning model (with extension .xmodel that is generated in phase 5 of the implementation flow) into the DPU dedicated memory space; task instantiation and assignment; and encapsulating the calls to invoke the XRT. The VART runtime resorts to XRT, which interacts with the driver ZOCL (it also performs FPGA manager integration in fabric by fetching .xclbin file containing bitstream) for memory allocation to perform all the functionalities mentioned above as it offers a software interface between the application code and the DPU kernel.

Finally, the last package included in the PetaLinux project is the ROS package, which is incorporated as a set of meta layers to Yocto so that its recipes can be parsed/processed for AArch64 architecture and generated kernel distribution. This software will be responsible for enabling and managing the communication with devices external to our platform, i.e., LiDAR sensor. After including the VART, XRT, ZOCL, and ROS packages, as long as their software libraries and dependencies are built into our PetaLinux project, the software components of our platform are generated. This software contains all necessary booting components (FSBL and PMU firmware and U-Boot), Linux image, Executable and Linkable Format (ELF) files, and sysroot with VART, Vitis AI Library, XRT, ZOCL, and ROS software.

Once both phases 1 and 2 are complete, i.e., hardware and software components are generated, phase 3 of our implementation process flow depicted in Figure 4 sets off. During this step, the Vitis tool provided a specific solution consisting of a single platform that integrates both HW and SW components. Thus, the Vitis IDE works with the hardware design created with the Vivado tool (.xsa) and the software components built using the PetaLinux tool and provides an OpenCL execution model and traditional C/C++ compilation. With the generated platform that supports kernels, we can now add a domain (our operation system with the collection mentioned above of software drivers and tools, on which to build the application) and develop applications that use our DPU kernel to run on the PS side, available to the operating system generated by the PetaLinux tool and integrated into the platform generated on the previous step. Only a single Linux domain for the Cortex™-A53 processor cores has been added to our platform.

The output of phase 3 is a board-specific SD card image that is used during Phase 4 of the block diagram, depicted in Figure 4 to program it to a micro-SD card using software for flashing OS images to SD cards. Finally, Phase 5 of the implementation flow regards the tasks of training and adapting/compiling our model for on-boarding inference and leveraging the VART C++/Python API to call Vitis AI Runtime and Vitis AI Library (where several pre-and post-processing functions and neural networks algorithms, with full XRT support, are provided) for building an executable software able to load the DPU kernel and run the compiled deep learning model file (.xmodel extension file) on our target Xilinx platform. Phase 5 is covered in detail in the next section.

### 3.3. Onboard Object Detection Architecture

This section describes the process of deploying our model to the edge device presented in the previous section, corresponding to Phase 5 from Figure 4. Deploying a neural network model to a resource-constrained device requires multiple adjustments and transformations to the model described in Section 3.1. We begin by describing the methodology followed to adapt and compile out the network for the targeting device, where we included the steps of quantisation and compilation. Thereafter, we present the three best performing model architecture configurations. In the last subsection, the quantitative and qualitative analysis results of the deployable models are reported, along with a comparative study between the performance of the floating-point models and their counterpart integer (quantised) versions.

#### 3.3.1. Quantisation and Compilation Methodology

The complexity of deep learning models is usually accompanied by high compute and memory bandwidth challenges. Reducing this overhead ultimately leads to an increase in power efficiency and lower total power requirements. In addition to saving power during computation, lower bit-width compute also lowers the power needed for memory bandwidth, reduces model size and the model’s inference time.

**Figure 6:**
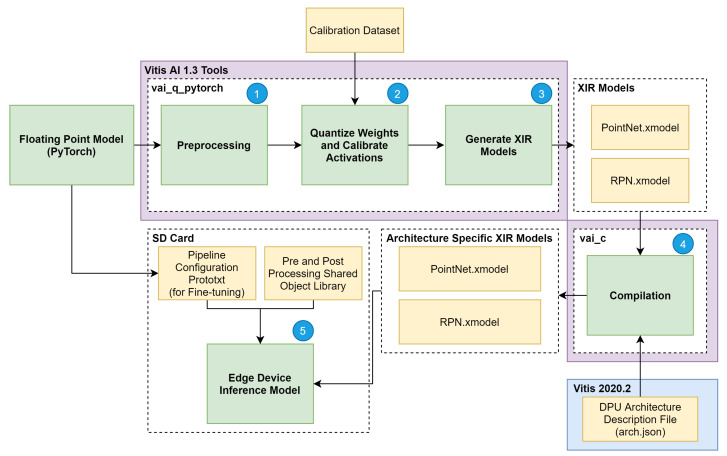
Model adaptation and optimisation based on post-training quantisation for on-board inference.

To ensure the low-latency and high-throughput requirements of the 3D object detection task on an edge device, a quantisation method can be applied to reduce the 32-bit floating-point weights and activations to an 8-bit integer format. Two different quantisations methods were followed in this article, namely post-training quantisation (PTQ) and quantisation aware training (QAT), and their results compared in terms of the onboard inference performance. The PTQ pipeline, which quantises an already trained model before converting the model to the Xilinx DPU instructions format, is presented in Figure 6. In **(1)**, PyTorch floating-point model and a PyTorch training checkpoint file are taken as input, and it is performed prepossessing on it, corresponding to the removal of useless model nodes and folding of the batch normalisation layers [28], resulting in a lighter and faster version of the model.

After the preprocessing stage, **(2)** the weights and activations of the model are quantised to a bit width of 8 (8-bit). To capture activation statistics and improve the accuracy of the quantised model, the Vitis AI quantiser requires a calibration dataset to perform calibration of the activations. This post-training calibration uses a cross-layer equalisation [29] which doesn’t require the calibration dataset to be labelled since no backpropagation is performed and only needs a small set of 100 to 1000 calibration examples to analyse the distribution of activations.

With the quantisation and calibration process complete, **(3)** the quantised model is transformed into a Xilinx Intermediate Representation (XIR) computational graph (xmodel). In this application, the model is split into two stages to distribute the computation between two DPU cores. The two outputted models are the PointNet.xmodel, corresponding to the Pillar Feature Net stage, and the RPN.xmodel, which includes both the backbone and detection heads.

The last step before deploying the model to the edge device **(4)** is to generate a compiled model based on the specified DPU microarchitecture. As shown in Figure 6, the compiler takes the output of the quantisation process (both XIR-based graphs) and a file describing the DPU architecture and generates two XIR-based graphs specifically for the desired target architecture. At this stage, the model is ready to be deployed **(5)**, by writing it to the SD card along with the shared object libraries containing the C++ implementation of the pre- and post-processing steps depicted in Figure 4 and pipeline management definition. On the other hand, the QAT approach applies quantisation during the training step of a model, simulating the expected low precision behaviour only in the forward pass of the training process. The quantisation error is thus accumulated in the loss of the model, forcing the optimiser to reduce the error outcome from the loss function by adjusting the parameters. This operation results in a set of more robust parameters, making the quantisation step almost lossless. Therefore, Steps (3), (4) and (5) in Figure 4 are also followed when applying the QAT approach to our model.

#### 3.3.2. Models Architecture

In this section, we selected the final network configuration for deployment on the edge device, considering that deploying to a resource-constrained environment will have costs on model performance. The candidate configurations resulting from the phase described in Section 3.1.1 will be subject to an evaluation to meet application requirements based on DPU configuration restrictions. The analysis of the DPU configuration selected and presented in Section 3.2.1 allow us to conclude that the impact on inference time of the inferior DPU capabilities compared to the reference of a highly paralleled system, such as a GPU, is expected to be relevant and can lead to exceeding the time requirements of 100 ms per inference step as further analysed.

**Figure 7:**
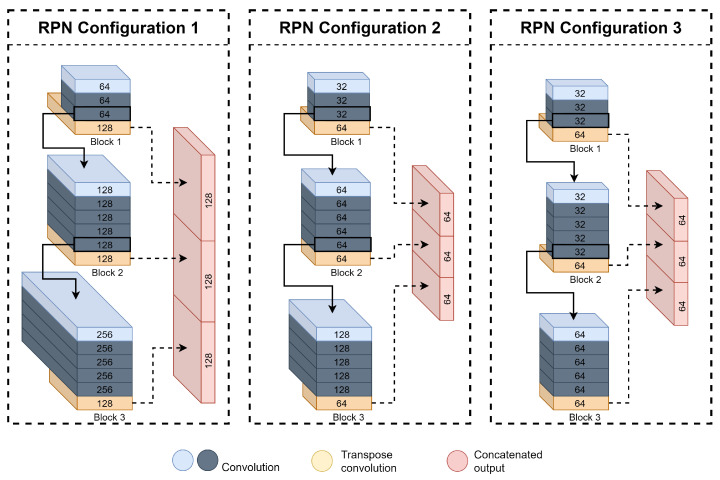
RPN configurations structure overview.

In order to reduce the inference time of the model while maintaining the general structure of the model architecture, a logical step is to lower the number of convolutions performed in the pipeline of the model proposed in Section 3.2.1. The RPN stage of the model, presented in Figure 7, is the most computationally complex stage of the pipeline and, consequently, bears the most considerable toll on processing time. Thus, due to the limited computation resources of the DPU configuration, we change the first block ($blc_{1}$) stride to two while maintaining the number of filters of the convolutional layers in the sequential blocks and the number of up-sampling filters of the transpose convolutions, according to Table 1. The resulted RPN configurations are shown below, in Figure 7, followed by a description of the resulting structure for each configuration in Table 11.

As demonstrated in Table 11, we only used three Detection Head configurations with stride 2 in $blc_{1}$ and according with configuration provided in Table 1. Although our *HDH* performs better in terms of AP results, its inference speed is impractical for real-time purposes and thus was discarded to this phase. The first configuration uses our *BDH*, where a higher number of filters is used. Configuration two uses our *IDH* version, where the number of filters is slightly lower than the *HDH* configuration. Finally, the third architecture, the lighter version, is used, namely *LDH* configuration, where the number of filters used is smaller when compared to the two versions mentioned before. Given the higher complexity of architecture one, it is expected to perform better in terms of precision, but due to its complexity, a penalty on the execution time is to be expected, as showcased in Table 12. In contrast, from architecture two, a balanced trade-off between inference time and precision, and finally, configuration three should be the fastest model configuration at the cost of detection quality. The impact of the changes on the RPN is discussed in the next section.

## 4. Results

In this section, the results of the previous steps are presented, comparing the floating-point and the two resulting integer versions of each configuration of the model presented in the previous subsection. These quantitative tests are performed using the KITTI Benchmark dataset. The results provided originate from evaluating the floating-point models and their pre-compilation quantised versions running on the server and the quantised models running on the edge device.

Two setups were used to perform model evaluation: server and edge device (Xilinx UltraScale+ MSoC ZCU102). The AArch64 Linux OS for our edge device was generated according to the process flow described in the previous section, while the DPU kernel was built and configured. The server setup used is composed of the following: Intel Core i9-10900K CPU; 64 GB RAM; Quadro RTX 6000 Graphic card; Ubuntu 18.04.5 LTS.

In Table 10 the performance of the different versions of the model regarding the configuration and data representation is presented. As can be seen, the time performance analysis was executed on both systems, extracting data from the floating-point models on the server and the quantised versions on both the server and the edge device.

As expected, the more complex configurations perform worse in terms of inference time. While the impact of the increased number of convolutions of Configuration 1 does not have a substantial impact on performance when running the model on the server-side (around 5 ms), due to the resource-constrained environment of the edge device, this difference increases massively, almost doubling the inference time of Configuration 1 and failing to meet the inference time project requirement of 100 ms as mentioned before. Both Configurations 2 and 3 achieve positive results surpassing the requirements by over 60% in inference speeds.

Table 13, Table 14 and Table 15 provide a set of results of the qualitative benchmark, containing information on the official KITTI evaluation detection metrics. The precision-based quantitative benchmarks for both the floating-point and quantised models were performed on the server setup.

From the evaluation results of the floating-point model configurations presented in these tables, it is clear that using an RPN structure with more convolutional filters achieves higher quality detection, especially for the smaller classes, such as cyclist and pedestrian, with a mAP increase reaching 8% for cyclist 3DBBOX in moderate difficulty when compared to the runner-up configuration. Configuration 1 achieves higher precision results over the other two configurations at the cost of more computational complexity.

For the AOS metric, this increase in detection performance is also evident, with gains of up to 6% over Configuration 2, and 11% over Configuration 3. The second-best performing model is Configuration 2, with a slight increase in precision over the simplest RPN of configuration 3. Again, this relative improvement is most apparent in the smaller classes and the 3DBBOX metric, with a 6% increase in hard difficulty for cyclists.

**Figure 8:**
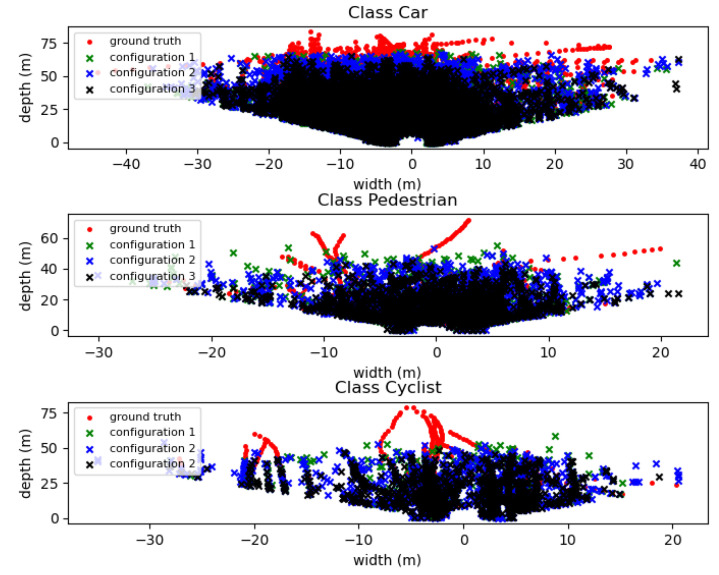
Representation of the location of the KITTI evaluation dataset’s ground truths and models predictions for all evaluation point clouds on a BEV perspective and according to the camera coordinate frame.

Changing the structure of the RPN for a simpler structure affects its performance, as it reduces the maximum object distance that the model is capable of detecting, in addition to localising it, the computed confidence score and the resulting IoU between ground truth and predicted objects. As depicted in Figure 8, where the location of the coordinates of all the KITTI dataset’s ground truth and predictions from models are displayed according to the coordinate frame, configuration 1 detects objects further away from the sensor than the others. Although it is almost unperceptive in Figure 8, configuration 2 performs slightly worst than configuration 1, but presents significant improvements over configuration 3 regarding maximum detectable object distance in a point cloud. This trend is also noticed for output confidence score and computed IoU, with configurations 1 providing higher values for both parameters (with confidence scores of 0.88, 0.78, 0.70 for car, cyclist and pedestrian, respectively, while computed IoU are respectively 0.86, 0.78, 0.66) than those of configuration 2 (confidence score 0.87, 0.74, 0.67, and IoU 0.86, 0.78, 0.65, respectively for car, cyclist and pedestrian) and configuration 3 (confidence score 0.85, 0.70, 0.66, and IoU 0.85, 0.77, 0.65, respectively). The confidence score parameter and the maximum distance of a detectable object decrease are more significant for small-size objects, such as cyclists and pedestrians, showing the loss of ability to learn resources at various scales of less complex RPN structures, indicating that the effect of the reduction in the number of layers on each block on the model performance is due to changes on the Shallow block. There is a significant loss of robustness in detecting small objects or large objects but quite far from the sensor due to the loss of spatially-rich information encoded during the processing of such small receptive fields with higher resolution. The operation of deep layers does not seem to be as strongly affected as it is during the processing of larger receptive fields, having a lower resolution, so that the required semantic-rich features are required to infer large objects, such as cars, or objects near to the sensors.

Figure 8 provides descriptive information about the PointPillars model behaviour. Although the point cloud range processed in terms of length is set to 0–69.12 m, most of the models, regardless of the complexity of the RPN, are unable to detect small objects, such as cyclists and pedestrians, at distances greater than 50 m from the sensor. This limitation is linked to the model performance and the low resolution of the point cloud, as the number of points representing objects is quite low. Therefore, we believe that this limitation should be seen as a restriction for applying the proposed model, and the precision performance assessment must consider it.

**Figure 9:**
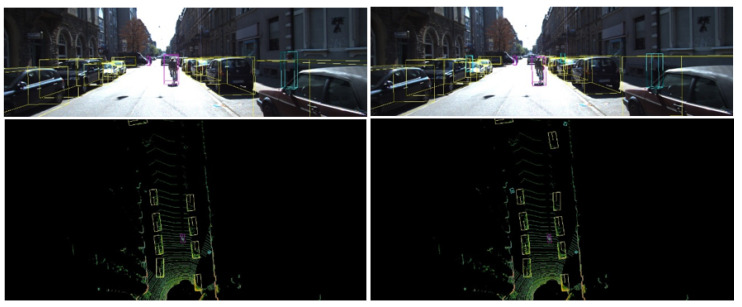
Example of a KITTI frame inference results for PTQ (left point cloud top view) and Optimised QAR (right image) configuration 2 model.

The results of the evaluation on the quantised model configurations, presented in Table 13, Table 14 and Table 15, show that the same trend is still present, with the most complex structure achieving higher results regardless of the type of adopted quantisation method. Though it is still clear that Configuration 1 results in higher precision than the other two, the overall gains are smaller in this PTQ quantised version of the model. It is also important to note the decay in detection quality resulting from the post-training quantisation process, with scores dropping around 10%, especially for the 3D bounding box metric. These results show the precision performance penalty due to reducing the resolution of the model parameters and input data. Although quantisation is inevitable, its influence on performance can be mitigated by considering the quantisation penalty on the model training step. QAT models show significant improvements, resulting in models that notably outperform their counterpart PTQ models, leading the resulting models to perform at the same level (sometimes even leads this benchmark) as their floating-point counterpart model, but with much lower inference times. Thanks to this quantisation process, it was possible to offer a solution that does need to sacrifice precision in favour of inference time, as the QAT solutions offer quite similar performance to their counterpart FP models. Figure 9 visually demonstrates the inference performance of both quantisation approaches, where it is clear that quantisation post-training reduces the model precision, weakening its capacity to handle the presence of small objects or objects far from the sensor in point cloud.

As expected, some predefined parameters, such as nms score threshold, strongly influence the model behaviour. In addition to the decrease in the average value found in models with less complexity, this value also decreases for quantised models. The best performing model, i.e., offering an optimal trade-off between precision and inference time, is selected and tested against different nms score thresholds.

The conducted study allows us to conclude that an nms score threshold of 0.4 enhances the model performance as showcased in Table 13, Table 14 and Table 15. The model called Optimised QAT follows the same configuration of its counterpart model at the exception of the updated nms score threshold and the point cloud range.

Therefore, previous observations were considered for adequately configuring the Optimised QAT models, namely maximum point cloud range (0–69.12 m for class Car, and 0–50 for cyclist and pedestrian) for class and nms score threshold (0.4 for all Classes).

Regarding our target platform’s energy consumption and resource usage, the DPU IP was configured to allow us to achieve an optimal balance between parallelism and resource usage while keeping the energy consumption low. Therefore, maximum parallelism provided by the target board was selected, i.e., up to 4096 Peak Ops; two cores adopted (one for each of the models found in the PointPillars pipeline); high usage of DSP blocks but low utilisation of BRAM blocks. This configuration reduces the number of digital resources significantly, as just 50% of the total o available BRAM blocks and DPU blocks are consumed, while Slice LUTs, and CLB registers usages is lower than 30%. The energy consumption for this DPU configuration is 15.7 W. Decreasing the number of adopted DPU cores leads to decreased energy consumption and resource usage of 5W and 30%, respectively, but an inference time penalty of 10 ms was reported. However, both configurations could be adopted given that the expected inference time is still lower than the threshold imposed by the application, providing us with the possibility to reduce inference time performance for reducing energy consumption and release resources that might be vital for another process also adopted in autonomous vehicles, such as SLAM.

## 5. Conclusions

Previous works in 3D Object detection for autonomous driving have been focused on improving the deep learning algorithms on the server-side with the main goal of improving metric precision without considering the requirements and restrictions of the real-case applications. Here, processing devices, so-called edge devices, offer much less computation power and memory resources. This research study focuses on optimising and compiling 3D Object Detection models for hybrid FPGA-CPU boards. It was shown that using the straightforward deployment of 3D object detection models in such resource-constrained devices is impractical. Therefore, the baseline model, the PointPillars, is adopted, and the most computing power demanding stages of its pipeline, i.e., CNN-based operations, as is the RPN case, were accelerated in hardware while preprocessing and post-processing stages are performed on the CPU part. The proposed methodology consists of a complete flow, which is three-fold. Firstly, network performance improvement via network configuration and hyperparameters optimisation is performed and discussed. Secondly, hardware and software required to execute Linux applications and DPU-related algorithms with enabled communications to the external devices, e.g., LiDAR scanners, are adequately configured in a way that offers an optimal balance between resource usage, processing speed and energy consumption. Finally, the performance results have been analysed, and required changes studied and applied. This methodology aimed to achieve an optimal balance between precision and inference-time at the same time that fulfils the inference time and energy consumption requirements of the targeted real-case application.

The PointPillars model was selected due to its trade-off between accuracy and inference time [4], and as the majority of related solutions, most of the computation stage is CNN-based. Moreover, its inference time margin permitted us to explore a fine-tuning process to increase accuracy while maintaining project requirements. Herein, we provided insights into the implication of several parameters in terms of accuracy gains and their impacts on the model inference time.

In this paper, a Zynq MPSoC device was adopted, and two compression techniques were applied to adjust the operations to the hardware limitations, namely pruning and quantisation. The later technique adjusted the arithmetic operations, converting weights, bias and input data for an 8-bit fixed-point representation. Both approaches drastically reduced inference speeds, making these complex models surpass the inference time requirements by over 60%. However, PTQ quantisation-based models reported scores dropping by approximately 10% in detecting objects from class cyclists and pedestrians. This performance penalty translated into the inability to detect small objects or any object located far from the sensor. The QAT approach showed notable performances that rival their counterpart floating-point model. The followed methodology displayed that the inference time can be reduced to acceptable values while maintaining notable performances for all object classes in a resource-constrained device that can easily be assembled in autonomous driving, with on-chip energy consumption low enough for vehicles and resource utilisation. At the same time, the performance of the models regarding energy consumption and resources used can be improved at the cost of the inference time, given the current difference between achieved inference time and threshold (40 ms). It might be helpful when more algorithms are expected to run on the same hardware device.

## Figures and Tables

**Table 1:** Detection Head filters and upsample filters configurations.

	*BLC*	Lighter	Intermediate	Baseline	Higher
	Number	(*LDH*)	(*IDH*)	(*BDH*)	(*HDH*)
***F***	$blc_{1}$	32	32	64	128
	$blc_{2}$	32	64	128	128
	$blc_{3}$	64	128	256	256
***U***	$blc_{1}$	64	64	128	256
	$blc_{2}$	64	64	128	256
	$blc_{3}$	64	64	128	256

**Table 2:** Number of sampling instances (*SI*) per class.

*SI* Configuration	Car	Pedestrian	Cyclist
$SI_{1}$	15	6	8
$SI_{2}$	15	15	15
$SI_{3}$	15	25	25

**Table 3:** The different point cloud ranges ($PC_{R}$) configurations used in fine-tuning.

${\mathrm{PC}}_{R}$ Configuration	$X_{\min}$	$X_{\max}$	$Y_{\min}$	$Y_{\max}$	$Z_{\min}$	$Z_{\max}$
$PC_{R1}$	0	69.12	−39.68	39.68	−3	1
$PC_{R2}$	0	70	−40	40	−2.5	1
$PC_{R3}$	0	52.8	−32	32	−3	1
$PC_{R4}$	0	47.36	−19.84	19.84	−2.5	0.5

**Table 4:** Pillar size ($S_{PL}$) configurations used in fine-tuning.

$S_{\mathrm{PL}}$ Configuration	$S_{\mathrm{PL}}\mathrm{length}$	$S_{\mathrm{PL}}\mathrm{height}$
$S_{PL16}$	0.16	0.16
$S_{PL25}$	0.25	0.25
$S_{PL5}$	0.05	0.05

**Table 5:** Total number of Pillars used in fine-tuning.

*P* Configuration	Total Number of Pillars	Max Number of Points Per Pillar
$P_{12K}$	12 K	100
$P_{30K}$	30 K	5

**Table 6:** The set of experiments conducted and respective network configurations.

Experiments	Detection Head Config.	${\mathrm{PC}}_{R}$ Config.	*SI* Config.	No. Output Classes	$S_{\mathrm{PL}}$ Config.	*P* Config.	No. Epochs
1	*LDH*	$PC_{R1}$	$SI_{1}$	3	$S_{PL16}$	$P_{12K}$	160
2	*BDH*	$PC_{R1}$	$SI_{1}$	3	$S_{PL16}$	$P_{12K}$	160
3	*IDH*	$PC_{R1}$	$SI_{1}$	3	$S_{PL16}$	$P_{12K}$	160
4	*HDH*	$PC_{R1}$	$SI_{1}$	3	$S_{PL16}$	$P_{12K}$	160
5	*BDH*	$PC_{R1}$	$SI_{2}$	3	$S_{PL16}$	$P_{12K}$	160
6	*IDH*	$PC_{R1}$	$SI_{3}$	3	$S_{PL16}$	$P_{12K}$	160
7	*BDH*	$PC_{R1}$	$SI_{2}$	3	$S_{PL16}$	$P_{12K}$	300
8	*BDH*	$PC_{R2}$	$SI_{1}$	3	$S_{PL25}$	$P_{12K}$	160
9	*BDH*	$PC_{R3}$	$SI_{1}$	3	$S_{PL5}$	$P_{30K}$	160
10	*BDH*	$PC_{R1}$	$SI_{2}$	3	$S_{PL16}$	$P_{12K}$	300
11	*IDH*	$PC_{R1}$	$SI_{2}$	3	$S_{PL16}$	$P_{12K}$	300
PointPillars (Car)	*BDH* (stride 2 in $blc_{1}$)	$PC_{R1}$	$SI_{1}$	1	$S_{PL16}$	$P_{12K}$	160
PointPillars (Pedestrian & Cyclist)	*BDH*	$PC_{R4}$	$SI_{1}$	2	$S_{PL16}$	$P_{12K}$	160

**Table 7:** Results in validation set for BEV detection metric.

Model/Experiment	Car			Cyclist			Pedestrian		
	Easy	Mod.	Hard	Easy	Mod.	Hard	Easy	Mod.	Hard
Experiment 1	90.15	82.78	82.7	78.41	63.86	59.53	57.18	52.08	47.07
Experiment 2	89.8	87.3	84.95	82.98	67.62	63.48	65.11	59.94	54.97
Experiment 3	89.71	87.27	85.11	82.28	64.81	60.54	61.01	55.28	50.13
Experiment 4	89.46	86.70	85.26	85.09	68.08	63.93	63.11	58.05	53.88
Experiment 5	89.23	86.52	84.49	69.13	53.47	49.57	65.29	58.85	53.05
Experiment 6	89.09	86.22	82.29	83.01	68.06	64.46	63.69	57.00	52.59
Experiment 7	89.94	87.26	85.56	82.85	66.85	62.60	62.93	57.13	53.11
Experiment 8	90.02	87.23	83.22	82.63	66.85	62.51	62.24	56.78	52.76
Experiment 9	89.80	76.69	68.30	78.70	59.36	58.16	66.75	59.63	52.55
Experiment 10	89.93	87.18	84.2	85.85	67.15	63.88	62.74	57.12	52.08
Experiment 11	90.02	87.65	85.83	83.25	66.85	62.25	59.83	54.37	50.30
PointPillars	89.74	86.05	81.65	82.47	62.79	59.52	68.23	63.58	59.83

**Table 8:** Results in validation set for 3D Bounding Box detection metric.

Model/Experiment	Car			Cyclist			Pedestrian		
	Easy	Mod.	Hard	Easy	Mod.	Hard	Easy	Mod.	Hard
Experiment 1	83.06	71.26	67.38	77.31	60.07	57.11	50.03	44.86	40.07
Experiment 2	85.62	76.41	71.98	79.54	64.28	60.87	57.04	52.73	47.88
Experiment 3	84.83	75.42	70.60	80.89	62.88	59.10	53.13	48.03	43.35
Experiment 4	84.47	76.51	73.28	83.36	64.68	61.41	54.81	49.71	45.6
Experiment 5	76.37	65.94	65.06	47.47	26.09	24.68	51.35	46.39	41.26
Experiment 6	80.25	73.66	70.13	80.34	63.69	60.25	56.28	50.96	46.36
Experiment 7	81.89	75.65	71.06	81.71	62.45	59.71	55.11	49.22	45.28
Experiment 8	81.35	74.88	69.28	79.08	62.43	59.45	51.05	46.45	42.94
Experiment 9	84.33	66.49	59.73	76.76	57.58	53.83	51.13	48.43	43.05
Experiment 10	87.12	77.04	74.33	84.47	63.86	61.73	55.65	50.42	45.81
Experiment 11	85.22	75.49	70.64	80.55	63.07	59.31	52.70	47.19	42.72
PointPillars	83.58	74.15	68.76	80.61	60.95	56.94	62.3	57.53	52.51

**Table 9:** Results in validation set for AOS detection metric.

Model/Experiment	Car			Cyclist			Pedestrian		
	Easy	Mod.	Hard	Easy	Mod.	Hard	Easy	Mod.	Hard
Experiment 1	90.25	86.14	80.96	75.28	61.32	57.87	30.8	28.55	26.54
Experiment 2	90.59	88.44	86.62	84.57	70.33	68.1	48.42	46.03	42.65
Experiment 3	90.29	87.91	85.87	79.29	62.86	59.64	33.07	30.9	28.94
Experiment 4	90.37	88.38	87.2	86.03	68.26	64.9	46.84	44.31	41.82
Experiment 5	89.72	81.09	80.61	51.93	29.7	28.15	35.95	34.01	31.46
Experiment 6	90.17	87.90	85.99	83.78	71.32	68.74	47.44	43.68	40.86
Experiment 7	90.38	87.81	86.23	82.21	65.31	62.38	54.07	50.97	47.98
Experiment 8	90.51	88.31	86.17	83.6	68.12	64.55	42.18	39.42	36.87
Experiment 9	90.16	79.03	68.88	78.17	58.55	57.83	40.34	37.9	36.42
Experiment 10	90.39	88.64	86.9	85.85	66.78	64.53	54.31	50.84	47.7
Experiment 11	90.46	88.20	85.99	83.37	67.19	63.76	46.85	44.32	41.66
PointPillars	90.51	88.2	86.01	82.23	62.48	59.28	34.27	33.2	31.95

**Table 10:** Inference time metric benchmark results.

Model/Experiment	Total (ms) ≈	Speed (Hz) ≈
Experiment 1	75.543	13.237
Experiment 2	94.488	10.583
Experiment 3	83.162	12.025
Experiment 4	127.666	7.833
Experiment 5	88.31	11.324
Experiment 6	82.063	12.186
Experiment 7	89.905	11.123
Experiment 8	33.453	29.893
Experiment 9	499.908	2.000
Experiment 10	104.1	9.606
Experiment 11	79.687	12.549
PointPillars (Car)	23.294	42.929
PoinPillars (Pedestrian & Cyclist)	27.48	36.390

**Table 11:** The set of network configurations used.

Config.	Det. Head Config.	${\mathrm{PC}}_{R}$ Config.	*SI* Config.	No. Out. Class.	$S_{\mathrm{PL}}$ Config.	*P* Config.	No. Epochs
1	*BDH*	$PC_{R1}$	$SI_{1}$	3	$S_{PL16}$	$P_{12K}$	300
2	*IDH*	$PC_{R1}$	$SI_{1}$	3	$S_{PL16}$	$P_{12K}$	300
3	*LDH*	$PC_{R1}$	$SI_{1}$	3	$S_{PL16}$	$P_{12K}$	300

**Table 12:** Inference time metric benchmark results, given in Hz, for floating-point and quantised models running in different machines.

Configuration	Floating-Point (Server)	PTQ (Server)	PTQ (Edge Device)	QAR (Edge Device)
1	21.2	23.5	9.5	9.4
2	23.2	26.1	16.6	16.7
3	28.2	26.0	18.7	18.7

**Table 13:** Results of the floating-point and quantised models on KITTI BEV detection for classes Car (IoU 0.70), Cyclist (IoU 0.50), and Pedestrian (IoU 0.50).

Config.	Version	Car			Cyclist			Pedestrain		
		Easy	Mod.	Hard	Easy	Mod.	Hard	Easy	Mod.	Hard
1	Float P.	89.64	87.30	79.42	78.44	60.72	55.31	52.98	50.75	44.74
	PTQ	89.44	86.62	79.04	73.07	53.81	51.99	49.12	43.46	42.19
	QAT	89.76	87.50	79.48	81.32	63.02	56.93	56.93	50.91	44.70
2	Float P.	89.75	87.22	79.35	72.49	53.75	52.14	59.98	53.75	52.14
	PTQ	88.82	85.51	77.93	67.14	52.27	47.66	48.91	46.61	41.02
	QAT	89.72	86.93	79.07	70.93	58.08	52.65	51.28	45.36	43.39
	**Optimised QAT**	89.72	86.93	79.15	73.97	61.00	56.38	54.22	49.16	46.34
3	Float P.	89.85	86.94	79.24	71.13	51.80	49.85	51.45	45.13	38.74
	PTQ	89.18	79.1	78.24	69.65	49.87	44.72	50.27	43.79	43.26
	QAT	89.85	86.94	79.24	70.24	52.13	47.61	50.43	44.89	42.49

**Table 14:** Results of the floating-point and quantised models on KITTI 3D BBOX detection.

Config.	Version	Car			Cyclist			Pedestrain		
		Easy	Mod.	Hard	Easy	Mod.	Hard	Easy	Mod.	Hard
1	Float P.	84.3	74.82	67.74	73.04	60.06	54.66	47.51	42.07	36.22
	PTQ	82.11	66.72	65.31	68.43	49.3	45.22	41.32	38.9	33.7
	QAT	85.09	75.46	68.19	72.22	55.53	54.52	48.86	46.91	41.05
2	Float P.	83.68	68.24	66.46	71.28	52.26	50.68	46.26	40.34	34.90
	PTQ	73.97	64.27	62.31	63.55	46.8	45.13	43.67	38.04	33.65
	QAT	78.57	68.14	66.23	69.18	56.41	51.29	46.32	40.89	35.09
	**Optimised QAT**	83.39	73.10	66.28	71.62	56.72	54.19	45.78	43.40	37.75
3	Float P.	77.44	67.36	65.78	74.87	53.00	51.25	44.61	39.36	34.26
	PTQ	72.56	62.31	55.49	64.83	47.94	43.05	37.29	32.92	32.3
	QAT	77.44	67.36	65.78	74.87	53	51.25	44.61	39.36	34.26

**Table 15:** Results of the floating-point and quantised models on KITTI AOS detection.

Config.	Version	Car			Cyclist			Pedestrain		
		Easy	Mod.	Hard	Easy	Mod.	Hard	Easy	Mod.	Hard
1	Float P.	90.68	88.29	79.85	76.5	60.21	54.7	32.63	29.08	28.81
	PTQ	89.97	86.86	78.76	68.1	51.47	50.15	28.1	26.05	26.17
	QAT	90.59	88.51	79.95	78.52	61.37	55.31	34.18	34.06	30.45
2	Float P.	90.48	87.70	79.50	68.44	56.08	50.78	27.35	25.26	22.51
	PTQ	89.23	85.7	77.5	66.67	53.08	48.78	24.87	22.12	21.2
	QAT	90.49	87.41	79.35	70.49	55.63	54.2	30.16	28	27.47
	**Optimised QAT**	90.50	87.35	86.05	69.90	58.29	54.26	31.45	29.95	29.17
3	Float P.	90.42	87.52	79.38	71.85	54.27	50.7	31.2	28.21	26.23
	PTQ	90.24	86.71	78.51	65.51	48.83	46.83	21.45	21.09	17.47
	QAT	90.42	87.46	79.4	72.95	55.27	50.7	29.8	27.06	26.55
